# Quantifying lumbar vertebral perfusion by a Tofts model on DCE-MRI using segmental versus aortic arterial input function

**DOI:** 10.1038/s41598-021-82300-6

**Published:** 2021-02-03

**Authors:** Yi-Jui Liu, Hou-Ting Yang, Melissa Min-Szu Yao, Shao-Chieh Lin, Der-Yang Cho, Wu-Chung Shen, Chun-Jung Juan, Wing P. Chan

**Affiliations:** 1 Department of Automatic Control Engineering, Feng Chia University, Taichung, Taiwan; 2 Master’s Program of Biomedical Informatics and Biomedical Engineering, Feng Chia University, Taichung, Taiwan; 3 Ph.D. Program in Electrical and Communication Engineering in Feng Chia University, Taichung, Taiwan; 4 Department of Nuclear Medicine, Chang Gung Memorial Hospital, Keelung, Taiwan; 5 Department of Radiology, Wan Fang Hospital, Taipei Medical University, 111 Hsing-Long Road, Section 3, Taipei, 116 Taiwan; 6 Department of Radiology, School of Medicine, College of Medicine, Taipei Medical University, Taipei, Taiwan; 7 Department of Neurosurgery, China Medical University Hospital, Taichung, Taiwan; 8 Department of Radiology, School of Medicine, College of Medicine, China Medical University, Taichung, Taiwan; 9 Department of Medical Imaging, China Medical University Hospital, Taichung, Taiwan; 10 Department of Medical Imaging, China Medical University Hsinchu Hospital, Hsinchu, 199, Sec. 1, Xinglong Rd., Zhubei City, Hsinchu County 302 Taiwan

**Keywords:** Biomedical engineering, Blood flow, Medical research, Diagnostic markers

## Abstract

The purpose of this study was to investigate the influence of arterial input function (AIF) selection on the quantification of vertebral perfusion using axial dynamic contrast-enhanced magnetic resonance imaging (DCE-MRI). In this study, axial DCE-MRI was performed on 2 vertebrae in each of eight healthy volunteers (mean age, 36.9 years; 5 men) using a 1.5-T scanner. The pharmacokinetic parameters *K*^*trans*^, *v*_*e*_, and *v*_*p*_, derived using a Tofts model on axial DCE-MRI of the lumbar vertebrae, were evaluated using various AIFs: the population-based aortic AIF (AIF_PA), a patient-specific aortic AIF (AIF_A) and a patient-specific segmental arterial AIF (AIF_SA). Additionally, peaks and delay times were changed to simulate the effects of various AIFs on the calculation of perfusion parameters. Nonparametric analyses including the Wilcoxon signed rank test and the Kruskal–Wallis test with a Dunn–Bonferroni post hoc analysis were performed. In simulation, *K*^*trans*^ and *v*_*e*_ increased as the peak in the AIF decreased, but *v*_*p*_ increased when delay time in the AIF increased. In humans, the estimated *K*^*trans*^ and *v*_*e*_ were significantly smaller using AIF_A compared to AIF_SA no matter the computation style (pixel-wise or region-of-interest based). Both these perfusion parameters were significantly greater using AIF_SA compared to AIF_A.

## Introduction

Dynamic contrast-enhanced magnetic resonance imaging (DCE-MRI) exploits tissue perfusion properties via quantitative analysis using appropriate tracer kinetics models^1^. Bone perfusion involves a blood circulation process which comprises several factors such as blood flow, capillary capacitance, permeability, interstitial space volume, interstitial diffusion, and venous return^2^.

Since 1991, DCE-MRI has been widely used in vivo. For example, bone marrow perfusion of the proximal femur was evaluated in a dog model by Cova et al.^3^. In 1995, Fujisawa et al. demonstrated vascular ingrowth after surgery in avascular necrosis of the femoral head^4^. In tumor studies, the perfusion of benign and malignant lesions in bone marrow^5–8^ and in lymphoproliferative diseases with diffuse bone marrow involvement^9^ has been investigated, and responses to chemotherapy have been monitored^10^. In the human spine, disc degeneration has been correlated with low vertebral perfusion, and an inverse association between vertebral perfusion and age has been demonstrated^11^. Maximal vertebral enhancement and the enhancement slope have been shown to decrease in those with osteopenia or osteoporosis^12,13^. Moreover, DCE-MRI outperforms conventional MRI in discriminating hypervascular from hypovascular spinal metastases^14^.

A two-compartment model has been most often used for quantitative analyses measuring the vertebral marrow perfusion. A tracer kinetic analysis^1^ produces four perfusion parameters: *K*^*trans*^, the efflux rate of gadolinium contrast from blood plasma to the extracellular extravascular space (EES); *K*_*ep*_, the rate constant for the return from the EES into the blood plasma; *v*_*e*_, the extracellular extravascular volume fraction; and *v*_*p*_, the volume fraction. Arterial input function (AIF) obtained from the tracer time curve for the aorta has been applied in quantitative perfusion models used in vertebral studies^15–17^. Whether the aorta is the only choice for AIF in vertebral perfusion studies is debatable. In humans, each vertebral body is supplied directly by a pair of segmental arteries arising from the aorta^18,19^. It is plausible that AIF obtained from the patient-specific segmental artery (AIF_SA) is less biased than AIF obtained from the patient-specific aorta (AIF_A) when using DCE-MRI for quantifying vertebral perfusion. To the best of our knowledge, lumbar perfusion using AIF_SA on DCE-MRI has not yet been documented.

We hypothesized that the amplitude and phase of AIF_SA differ from those of AIF_A and that perfusion parameters computed based on AIF_SA differ from those based on AIF_A. The aim of this study was to verify the effects of AIF amplitude and phase on perfusion parameters in simulation and to examine the differences in lumbar vertebral perfusion parameters computed based on the AIF_SA compared to AIF_A using axial DCE-MRI.

## Materials and methods

All experiments and methods were carried out in compliance with relevant guidelines and regulations. All experimental protocols were approved by the Institutional Review Board of Wan Fang Hospital. Written informed consent was obtained from each participant. A total of eight healthy volunteers (mean age 36.9 years; range 23–64 years; 5 male) were enrolled in this prospective study. Enrollment was limited to eight to minimize risks associated with the intravenous injection of gadolinium-based contrast agent, as mentioned elsewhere^20^.

### MRI protocols

**Figure 1:**
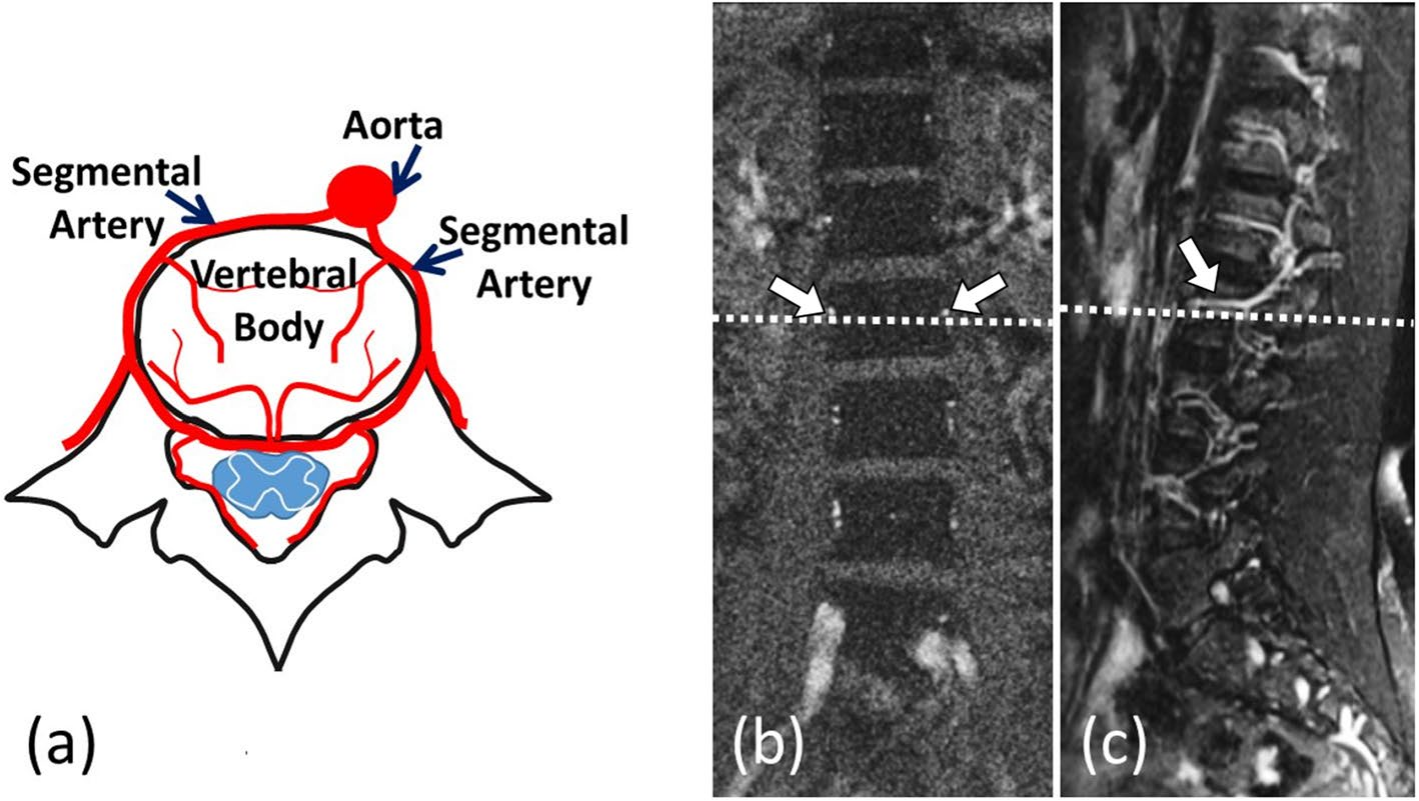
(**a**) The anatomic relationships between a lumbar vertebral body and its supplying arteries, including the abdominal aorta and segmental arteries. The axial plane through the L1 segmental arteries (just above the dotted lines) was selected on coronal (**b**) and sagittal (**c**) images of magnetic resonance angiography. White arrows indicate the segmental arteries.

All MR scans were performed using a clinical whole-body scanner with 1.5-T magnetic field strength (Avanto, Siemens, Erlangen, Germany). To identify the location of the segmental arteries, a 3-D TrueFISP non-contrast magnetic resonance angiography was acquired on the coronal plane covering the entire vertebral body using the following parameters: slice thickness, 2 mm; field-of-view, 360 × 360 mm; matrix size, 256 × 256; repetition time(TR)/echo time (TE), 508.9 ms/1.5 ms; and flip angle, 80°. Two axial planes of the lumbar vertebrae along the course of the segmental arteries at L1 and L2 were identified (Fig. 1). Then, axial T1 measures and DCE-MRI were performed, applying identical settings for field-of-view (30 × 30 cm), matrix size (256 × 250), and slice thickness (10 mm). Gradient echo images using a fast low angle shot (FLASH) sequence with a TR/TE of 19 ms/4 ms, and flip angles of 5°, 10°, 20°, 40°, 60°, and 80° were acquired to calculate T1. Using another FLASH sequence at the same TR/TE but a flip angle of 40°, another DCE-MRI was acquired, achieving more T1-weighted images via gradient echo sequences and obtaining a greater SNR for measuring T1 compared to what could be obtained using a smaller flip angle. A parallel imaging technique (GeneRalized Autocalibrating Partially Parallel Acquisitions; GRAPPA) was used, applying to dynamic scans an acceleration factor of 2 and a partial Fourier acquisition along the phase encoding direction with a factor of 6/8, netting 1.8 s (19 ms × 250 × (6/8)/2) to complete a dynamic scan. The temporal resolution was 2 s when two axial slices were acquired within one TR interval. Total acquisition time was 7 min and 20 s. A pre-saturation band with parallel slices was applied to the upstream aorta to eliminate the in-flow pulsation. An auto-injector was used to administer a bolus injection of Gd-DOTA (total dose, 0.1 mmol/kg) followed by a 10-ml saline flush via an arm vein at a rate of 2 ml/s.

### Data analysis

#### MR signal to concentration of contrast agent

**Figure 2:**
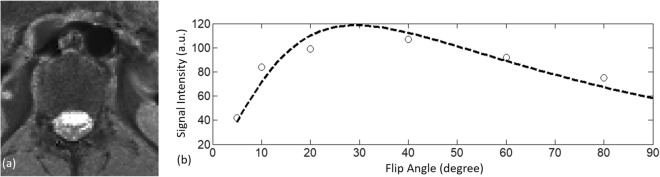
A T1 map (**a**) was generated from a pixel-by-pixel curve fitting of a signal-intensity-flip-angle curve (**b**).

Post-processing of the simulated data and MRI images was performed using in-house software implemented in MATLAB (The MathWorks Inc., Natick, MA), then T1 maps were calculated using the variable flip angle T1 measures (Fig. 2)^21^. The signal of a gradient echo using a spoiled gradient is:

1 $$S = \frac{{M_{0} \sin \alpha \left( {1 - e^{{ - TR/T_{1} }} } \right)}}{{1 - \cos \alpha e^{{ - TR/T_{1} }} }}{ }e^{{ - TE/T_{2}^{*} }} ,$$

where M_0_ is the equilibrium magnetization, and α is the excitation flip angle. The concentration of contrast agent was transformed from the MR signal based on the T1 relaxation rate:

2 $${\text{C}}\left( {\text{t}} \right) = \frac{1}{{r_{1} }}\left( { \frac{1}{{T_{1} \left( t \right)}} - \frac{1}{{T_{10} }}} \right),$$

where *C*(*t*) is the concentration of the contrast agent during DCE-MRI, r_1_ is the relaxation rate for the contrast agent (3.6 s^−1^ mmol^−1^)^22^, *T*_1_(*t*) is the T1 value during DCE-MRI, and *T*_10_ is the pre-contrast T1 value, as obtained in Eq. (1). Based on Eq. (1), the relationships between the dynamic MR signal, the initial MR signal, and the T1 relaxation rate in a gradient echo with a spoiled gradient is:

3 $$\frac{S\left( t \right)}{{S_{o} }} = \frac{{\left( {1 - e^{{ - TR/T_{1} \left( t \right)}} } \right)}}{{1 - \cos \alpha e^{{ - TR/T_{1} \left( t \right) }} }} \frac{{1 - \cos \alpha e^{{ - TR/T_{10} }} }}{{\left( {1 - e^{{ - TR/T_{10} }} } \right)}}.$$

Theoretically, because the time-invariant terms including ($M_{0} \sin \alpha$) and ($e^{{ - TE/T_{2}^{*} }}$) in Eq. (1) were cancelled, the signal ratio (${{S\left( t \right)} \mathord{\left/ {\vphantom {{S\left( t \right)} {S_{o} }}} \right. \kern-\nulldelimiterspace} {S_{o} }}$) will be less influenced by the T2* term. Therefore, the concentration of the contrast agent can be calculated by substituting of *T*_10_ and *T*_1_(*t*), obtained from Eqs. (1) and (3), into Eq. (2).

#### Pharmacokinetic modelling

In this study, the analysis of the DCE-MRI data was based on a modified Tofts model^23,24^ that assumes the contrast media reaches an equilibrium between the plasma and the EES via an iso-directional permeability described below:

4 $$C_{t} \left( {\text{t}} \right) = v_{p} C_{p} \left( {\text{t}} \right) + C_{p} \left( {\text{t}} \right) \otimes K^{trans} {\text{exp}}\left( { - K^{trans} t/v_{e} } \right),$$

where *C*_*t*_(*t*) denotes the contrast concentration in tissue, *v*_*p*_ denotes the fractional plasma volume, *C*_*p*_(*t*) denotes the contrast concentration in the plasma for the AIF, *K*^*trans*^ denotes the transfer constant (from plasma into EES), and *v*_*e*_ denotes the fractional volumes of EES per unit volume of tissue. Note that *K*^*trans*^/*v*_*e*_ = *k*_*ep*_, the reflux rate, and the plasma concentration of the AIF (*C*_*p*_) is calculated as the concentration time curve of AIF (*C*_*b*_(*t*)) multiplied by 1/(1 − Hct), where Hct (hematocrit) is 0.42^25^.

Vertebral perfusion was quantified on a pixel-by-pixel basis using regions of interest (ROIs) that contour the entire vertebral body and arteries selected for calculating AIF, respectively. Perfusion parametric maps were constructed using a deconvolution method based on a nonlinear least-squares fitting^26–29^. Perfusion parametric maps of the vertebra constructed by using AIF_A and AIF_SA were generated based on a pixel-wise method (PWM) and an ROI-based method (RBM) for comparison.

#### AIF selection

**Figure 3:**
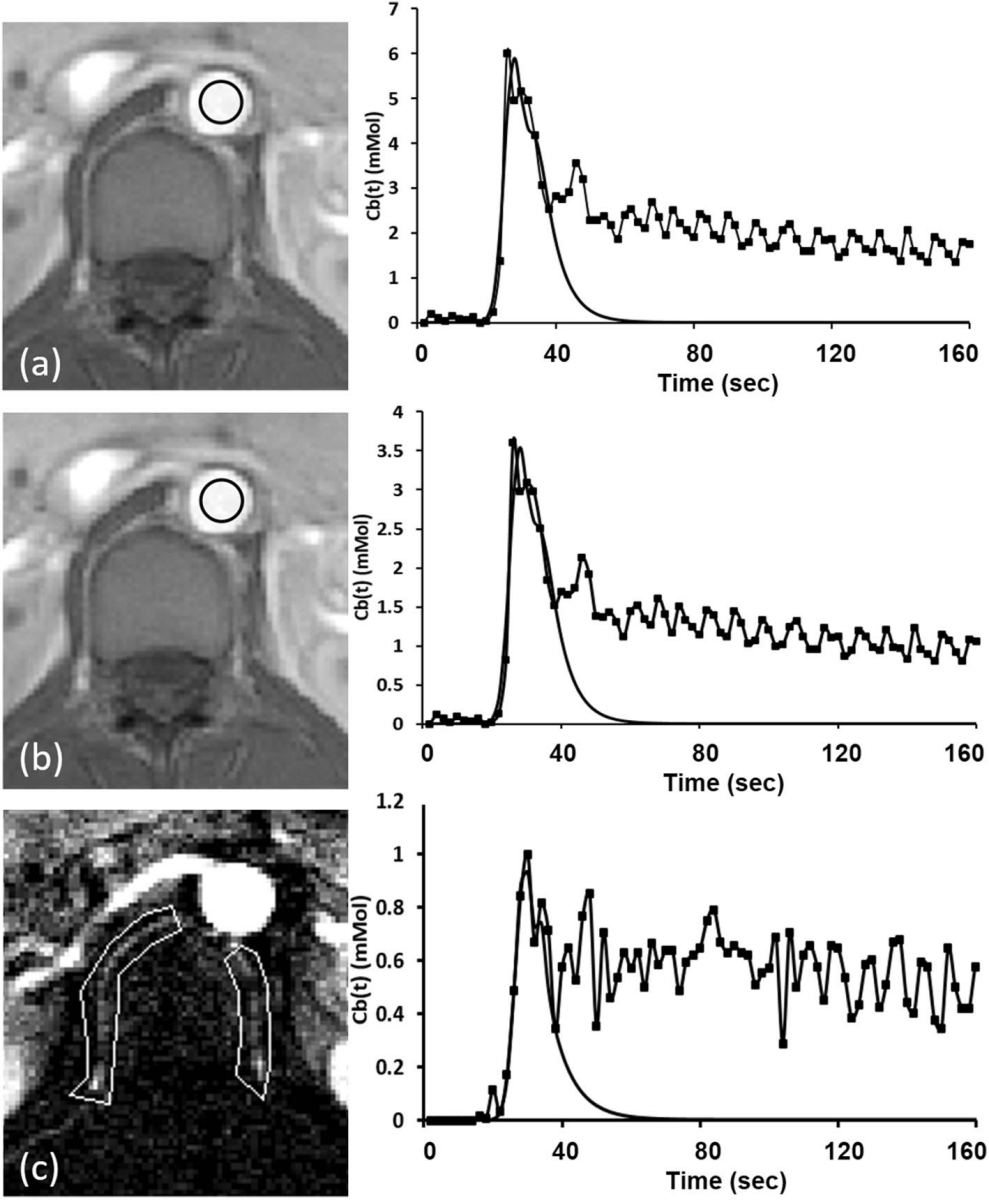
Selecting the region of interest for the aorta, using the peak contrast concentration in the population blood (**a**) or the individual blood concentration (**b**) and for the segmental arteries (**c**). Each is accompanied by its mean concentration time curves (solid line; squares indicate data points) and arterial input functions (solid line alone), fitted using a mixture between a Gaussian function and an exponential function modulated by a sigmoid function.

The manually selected ROIs of the segmental arteries and aorta together with their corresponding concentration time curves and curve-fitted AIFs are shown in Fig. 3. To minimize the influence of the partial volume effects on the segmental artery, subtracted dynamic images were generated by subtracting each dynamic image from the average across the first 5 phases of the dynamic images, obviously highlighting and clearly distinguishing the segmental arteries from the surrounding static tissue (Fig. 3c). The segmental artery on either side was then segmented by manually contouring an ROI on the subtracted dynamic images, encompassing that artery. The signal time curve was plotted for each pixel within that ROI, and the pixel with the maximal peak value was automatically chosen. Finally, AIF_SA was computed from the concentration time curve of that pixel. On the other hand, AIF_A was computed from the concentration time curve of the aortic ROI, obtained by manually placing a circular ROI within the aorta. For comparison, the aortic time curve was transformed into a population-based time curve, from which the AIF_PA, also known as the Parker AIF (6 mmol in blood concentration)^25,30^, was generated, compensating for biases arising from high arterial concentrations of the contrast agent, inflow effects, and partial volume effects in the arterial AIF estimation (*C*_*b*_(t))^25,31^.

Equation (5), integrating a Gaussian function and an exponential function modulated by a sigmoid function, was applied for curve fitting the first-pass component of the AIF, eliminating the influences of the second-pass circulation and venous blood flow^25^.

5 $$C_{b} \left( t \right) = \frac{A}{{\sigma \sqrt {2\pi } }}e^{{\left( {\frac{{ - \left( {t - T} \right)^{2} }}{2\sigma }} \right)}} + \alpha \frac{{e^{ - \beta t} }}{{\left( {1 + e^{{ - s\left( {t - \tau } \right)}} } \right)}}$$

where *A*, *T*, and *σ* are the scaling constants, centers, and widths of the Gaussian function, respectively, *α* and *β* are the amplitudes and decay constants, respectively, of the exponential function, and *s* and *τ* are the width and center of the sigmoid function, respectively.

#### Calculation of signal-to-noise ratio (SNR) of AIF

The temporal SNR of the AIF is calculated according to the following equation:

6 $$SNR = \frac{{C_{max} }}{{C_{baseline\_SD} }}$$

where *C*_*max*_ represents the maximal value of the AIF and *C*_*baseline_SD*_ represents the standard deviation of the baseline of the AIF.

#### Simulations of AIFs with various peaks and time delays

**Figure 4:**
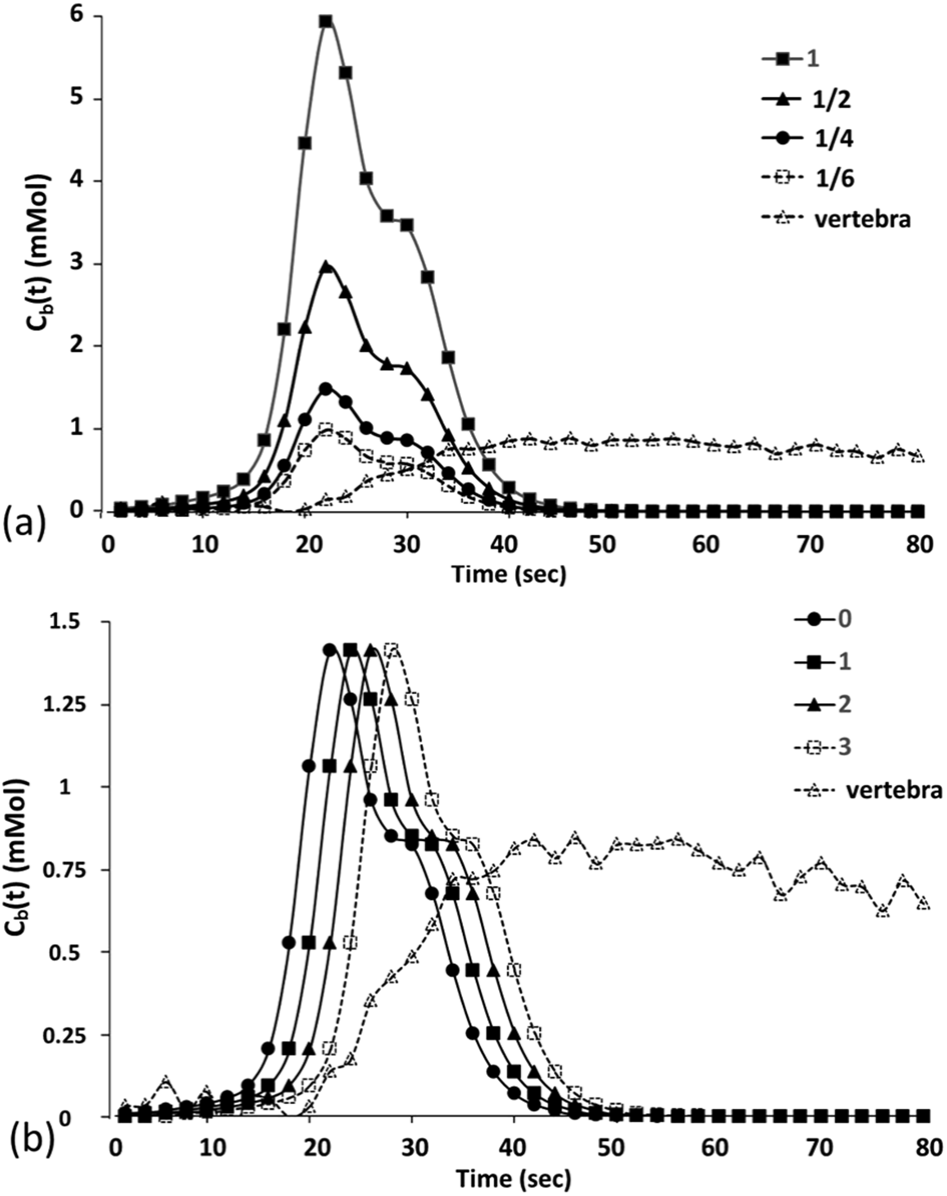
Simulation of arterial and vertebral concentration time curves generated using a typical aortic concentration time curve sample for one participant, showing the peak scaled by 1, 1/2, 1/4, and 1/6 (**a**) and the delay time adjusted by 0, 1, 2, and 3 time points (**b**). The first 80 of 440 s are shown.

By changing the peaks and delay times of a real aortic concentration time curve acquired from a given participant, AIFs were simulated to evaluate their influences on the perfusion parameters (*K*^*trans*^, *K*_*ep*_, *v*_*e*_, and *v*_*p*_). First, a typical AIF_A was generated from a chosen participant. Then, AIFs with various peaks were generated by rescaling the peak amplitude by factors of 1, 1/2, 1/4, and 1/6 (Fig. 4a). Similarly, AIFs with various delay times were generated by shifting the AIFs by 0, 1, 2, and 3 time points (Fig. 4b). A concentration time curve for the vertebra was acquired from the same participant, subsequently generating 1000 curves under each condition using the same SNR of 20 with random noise levels. Finally, the perfusion parameters from these 1000 curves were computed for each peak and delay times.

### Statistical analysis

Statistical analysis was performed by using SPSS software (IBM SPSS Statistics for Windows, Version 24.0; IBM Corp., Armonk, NY). The Wilcoxon signed rank test (WSRT) was used to compare the perfusion parameters between generated using a PWM and an RBM. The Mann–Whitney U test was used to compare the SNR of the concentration time curves of between the aorta and the segmental artery. The Kruskal–Wallis test (KWT) was used to compare the perfusion parameters generated using various AIFs and methods, applying a Dunn–Bonferroni post hoc correction across multiple comparisons. A *P* value less than 0.05 was considered to indicate statistical significance.

## Results

**Figure 5:**
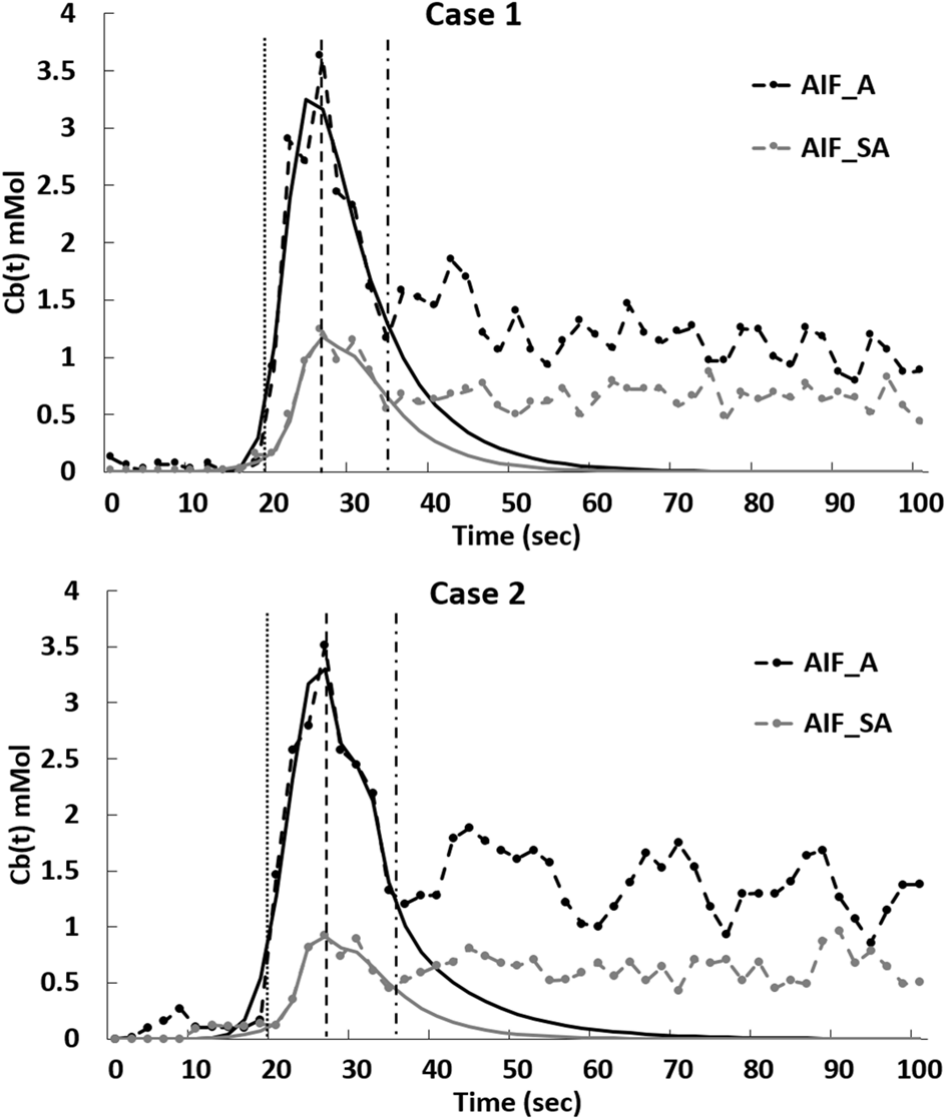
Concentration time curves and the corresponding first-pass fitted curves of the aorta and segmental artery of two participants. Dotted vertical lines represent the arrival of the first pass, dashed lines represent the peak, and dash-dotted lines represent the arrival of second pass of contrast-agent-containing blood. Black dashed curves represent the fit curves for the aortic arterial input function (AIF). Gray dashed curves represent the fit curves for the segmental arterial AIF.

The concentration time curves of the aorta and segmental arteries and the corresponding AIFs are shown in Fig. 5. The SNR (82.12 ± 30.81) of the aortic Cb(t) was significantly higher than that (37.94 ± 23.42) of the segmental arterial Cb(t) (*P* < 0.01). The transit time, defined as the interval between the arrival of the first pass and the arrival of second pass, remained consistent between the aortic and segmental arterial time curves. Data used for curve fitting the AIFs included the baseline, the upslope, and 3–4 data points after the peak in each concentration time curve, thus preventing contamination from the second pass (Fig. 5).

For every participant, the peak of AIF_A (5.04 ± 1.36 mmol; mean ± standard deviation) was significantly greater than the peak of AIF_SA (1.81 ± 0.53 mmol) (*P* < 0.001). No differences were found between contrast arrival times (aorta, 16.5 ± 2.33 s; segmental artery, 17.8 ± 2.47 s; *P* = 0.24).

**Figure 6:**
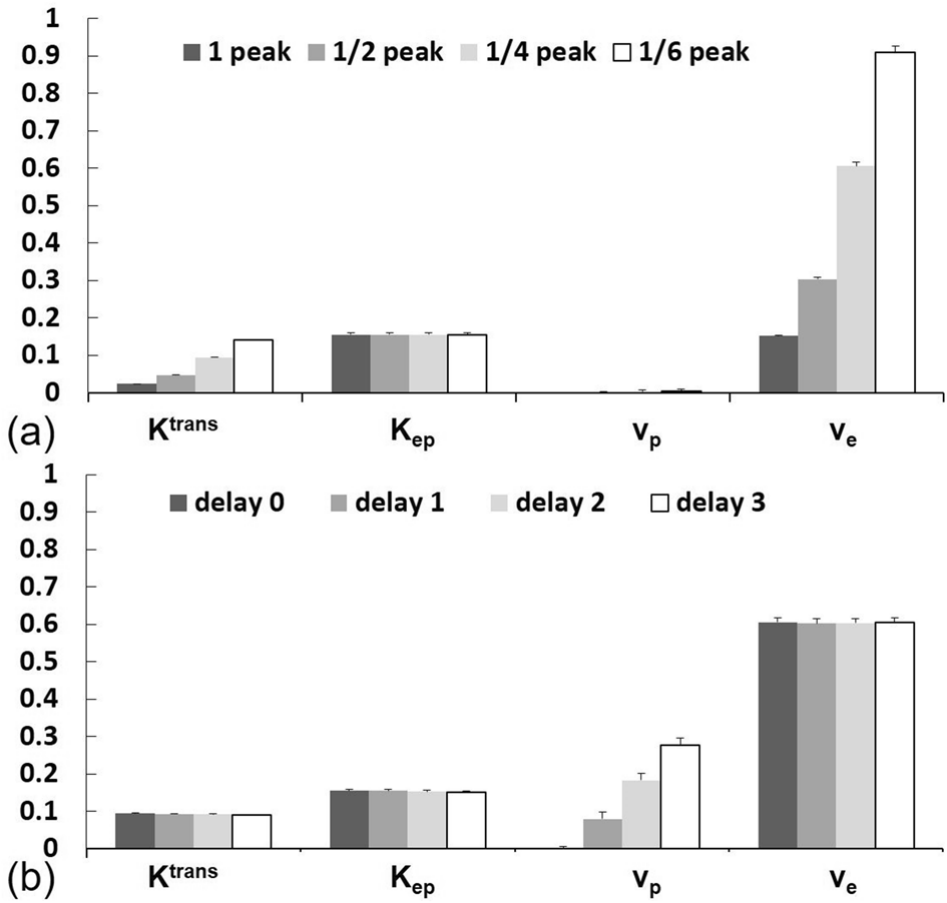
Perfusion parameters in a simulation study, varying (**a**) peaks and (**b**) delay times in the arterial input function.

Perfusion parameters at various AIF peak amplitudes and delay times in the simulation are shown in Fig. 6. As the peak amplitude of the AIF decreased, both *K*^*trans*^ and *v*_*e*_ obviously increased, but neither *K*_*ep*_ not *v*_*p*_ were affected. On the other hand, as the delay time of the AIF increased, *v*_*p*_ increased, but other perfusion parameters were not affected.

**Figure 7:**
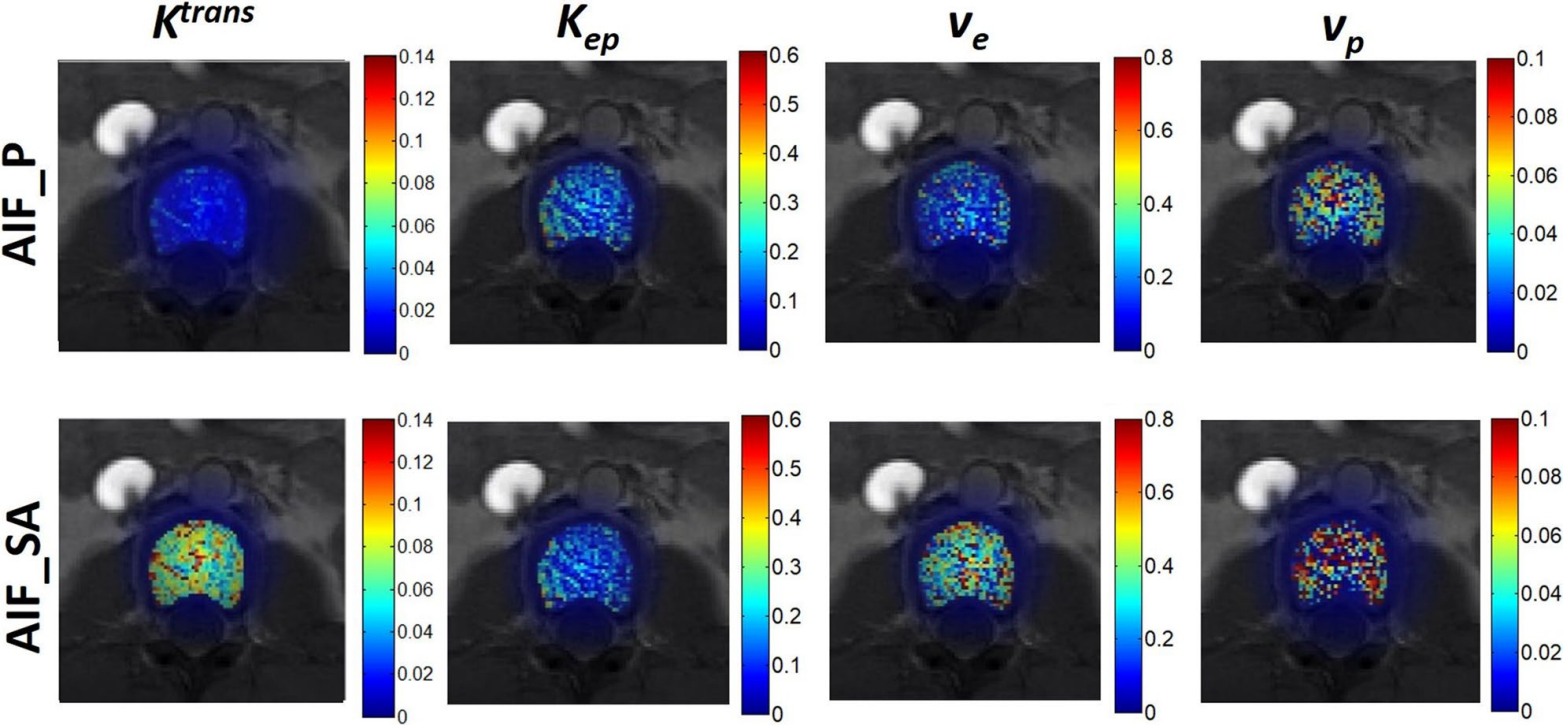
Maps of the perfusion parameters including *K*^*trans*^, *K*_*ep*_, *v*_*e*_, and *v*_*p*_ for one participant, using an aortic arterial input function (AIF; top row) or a segmental arterial AIF (bottom row).

Perfusion parametric maps calculated from AIF_A and from AIF_SA for a randomly selected participant are shown in Fig. 7. Both *K*^*trans*^ and *v*_*e*_ were obviously lower in the lumbar vertebra when the perfusion parameters are calculated using AIF_A compared to AIF_SA.

**Figure 8:**
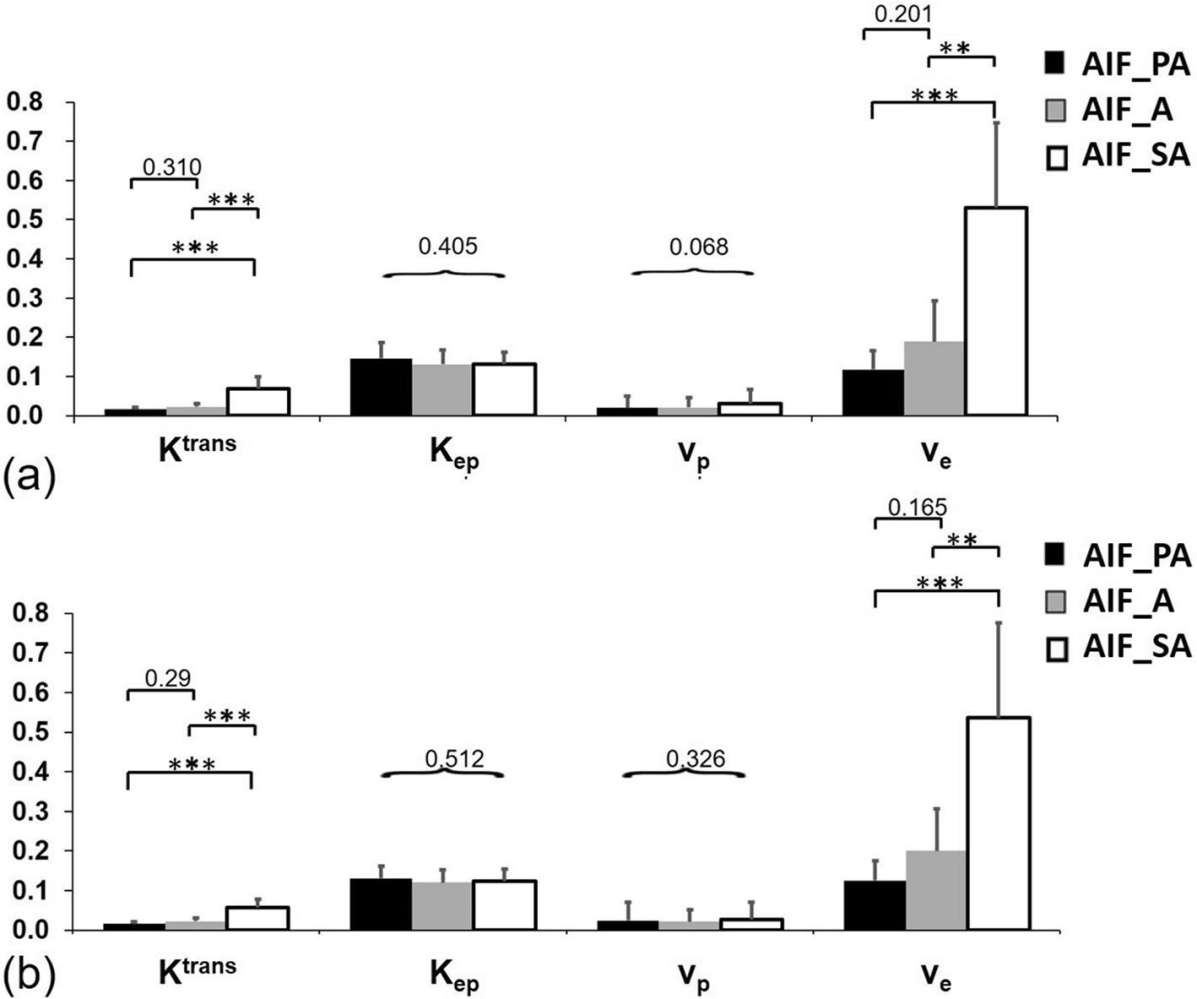
Comparing the perfusion parameters *K*^*trans*^, *K*_*ep*_, *v*_*e*_, and *v*_*p*_ between the population-based (Parker) arterial input function (AIF; black), aortic AIF (gray) and segmental arterial AIF (white) using (**a**) a pixel-by-pixel computational method and (**b**) a region-of-interest computational method. Note: data are presented as means and standard derivations. **P < 0.01, ***P < 0.001.

**Figure 9:**
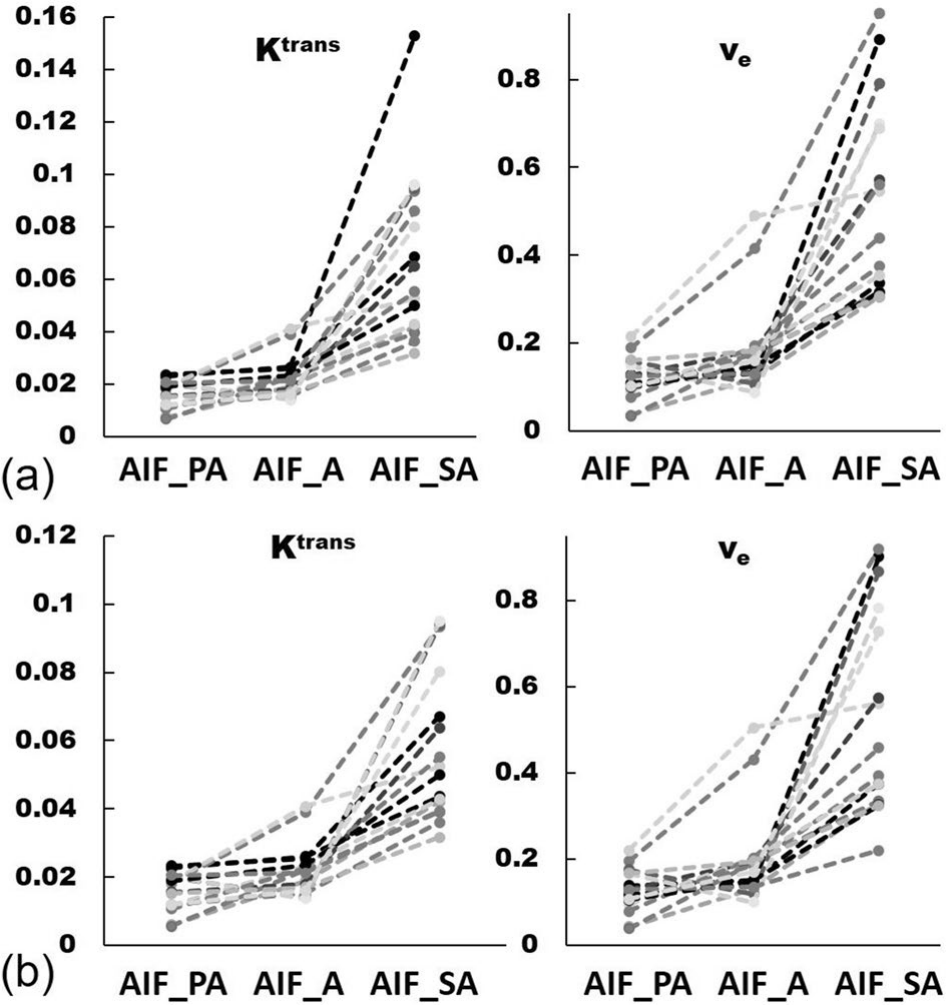
Values for *K*^*trans*^ and *v*_*e*_ calculated using the population-based (Parker) arterial input function (AIF), aortic AIF, and segmental arterial AIF in 16 vertebrae using a pixel-by-pixel method (**a**) and a region-of-interest method (**b**).

The perfusion parameters obtained using PWM and RBM are shown in Fig. 8. Values for *K*^*trans*^ using AIF_PA (0.0159 ± 0.0053 min^−1^ and 0.0156 ± 0.0055 min^−1^, respectively) and AIF_A (0.0225 ± 0.0079 min^−1^ and 0.0223 ± 0.0078 min^−1^, respectively) were significantly lower (*P* < 0.001) than those using the AIF_SA (0.0679 ± 0.0316 min^−1^ and 0.0578 ± 0.0219 min^−1^, respectively). Likewise, *v*_*e*_, determined using AIF_PA (0.1179 ± 0.0492 and 0.1253 ± 0.0509, respectively) as well as AIF_A (0.1888 ± 0.1083 and 0.2003 ± 0.1099, respectively) were significantly lower (*P* < 0.01) than those using AIF_SA (0.5296 ± 0.2241 and 0.5355 ± 0.2484, respectively). In particular, *K*^*trans*^ and *v*_*e*_, calculated using AIF_SA, were greater than those using AIF_PA and AIF_A, no matter the computation method applied (Fig. 9).

The WSRT indicated that *K*^*trans*^ and *K*_*ep*_, calculated using the PWM, were significantly greater than those calculated using the RBM (all *P* < 0.005), and that *v*_*e*_, calculated using the PWM, was significantly lower than that calculated using the RBM (all *P* < 0.01), no matter the AIFs selected. Finally, *v*_*p*_, *if* calculated using the PWM, was significantly greater than if calculated using the RBM (all *P* < 0.05), but only when AIF_SA was selected. On the other hand, the KWT did not indicate any significant differences between AIFs and computation methods when calculating any parameter except *v*_*p*_, which was significantly (*P* = 0.042) greater using the PWM vs. the RBM when AIF_SA was selected.

Comparisons of the perfusion parameters calculated using PWM and RBM are shown in Table 1. In all AIFs, the WSRT indicates that *K*^*trans*^ and *K*_*ep*_ calculated using PWM were significantly greater than those found using RBM (all *P* < 0.005), but that values for *v*_*e*_ calculated by using PWM were significantly lower than those found using RBM (all *P* < 0.01). Only for AIF_SA did *v*_*p*_ significantly differ: it was significantly greater using PWM compared to RBM (all *P* < 0.05). On the other hand, the KWT showed no significant differences in any parameter except for *v*_*p*_, which was significantly greater (*P* = 0.042) when PWM was used compared to RBM and only for AIF_SA.

**Table 1:** Comparison of perfusion parameters between PWM and RBM regarding different AIFs.

Parameters	Methods	AIF_P	AIF_A	AIF_SA
*K*_*trans*_ (min^−1^)	PWM	0.0159 ± 0.0053	0.0225 ± 0.0079	0.0679 ± 0.0316
	RBM	0.0156 ± 0.0055	0.0222 ± 0.0079	0.0578 ± 0.0219
	***P value***			
	WRST	***	***	***
	KWT	0.763	0.792	0.386
*K*_*ep*_ (min^−1^)	PWM	0.1462 ± 0.0418	0.1312 ± 0.0381	0.1314 ± 0.0320
	RBM	0.1310 ± 0.0314	0.1206 ± 0.0328	0.1244 ± 0.0308
	***P value***			
	WRST	***	***	***
	KWT	0.214	0.309	0.366
*v*_*p*_	PWM	0.200 ± 0.0308	0.0203 ± 0.0271	0.0314 ± 0.0358
	RBM	0.241 ± 0.0481	0.0219 ± 0.0314	0.0267 ± 0.0455
	***P value***			
	WRST	0.326	0.679	*
	KWT	0.065	0.083	*
*v*_*e*_	PWM	0.1179 ± 0.0493	0.1888 ± 0.1083	0.5296 ± 0.2242
	RBM	0.1253 ± 0.0509	0.2003 ± 0.1099	0.5355 ± 0.2485
	***P value***			
	WRST	***	***	**
	KWT	0.498	0.327	0.734

*P < 0.05, **P < 0.01, ***P < 0.001.

## Discussion

It is known that DCE-MRI is useful for detecting and characterizing tissue perfusion. The Tofts model is commonly used for quantitative analysis of DCE-MRI. To apply this model, AIF and the pre-contrast T1 value must be accurately measured^1^. However, determining the AIF remains technically challenging because of the location of the upstream, slice orientation, partial volume, and flow artifacts. Studies have shown that AIF can have a large effect on the accuracy of computed pharmacokinetic parameters^32–34^. Theoretically, a local AIF sampled at the inlet to the target tissue should be selected. In clinical practice, however, a major artery is often chosen instead, neglecting the effects of dispersion and delay of the AIF^32,35^. Allowing evaluation of multiple vertebrae in a single slice, AIF_A measured on the sagittal plane has been widely used in vertebral perfusion studies^8,15–17,36–39^. To the best of our knowledge, the effects of various AIFs on vertebral perfusion analysis has not yet been investigated.

In humans, each lumbar vertebra is supplied by a pair of segmental arteries which originate from the aorta^18,19^. This study showed that an axial slice allows the aorta and segmental arteries to be simultaneously scanned in each slice (Fig. 3) and therefore provides a chance to examine the effects of various AIFs (i.e., AIF_A and AIF_SA) on vertebral perfusion quantification. By intentionally perturbing the amplitude and delay time of an AIF, we simulated the effects of arterial size and location of an AIF on perfusion parameter quantification. Results showed that *K*^*trans*^ and *v*_*e*_ are associated with the peak of the AIF and that *v*_*p*_ is associated with AIF delay time (Fig. 6). Conceptually, AIF_SA has a smaller peak and somewhat longer time delay compared to AIF_A, obtained from the adjacent aorta. Therefore, it is expected that *K*^*trans*^ and *v*_*e*_, calculated using AIF_SA, would be greater than those found using AIF_A. As shown in Fig. 7, our perfusion parametric maps disclose relatively lower value for *K*^*trans*^, *v*_*e*_, and *v*_*p*_ in the lumbar vertebra when AIF_PA is used rather than AIF_SA. The results across 16 lumbar vertebrae in eight healthy people revealed similar trends in the simulation: *K*^*trans*^ and *v*_*e*_ were significantly greater when AIF_SA was used compared to AIF_A (Fig. 8).

The pros and cons of choosing axial slices in quantifying lumbar vertebral perfusion have not yet been explored. An axial slice allows AIF_A and AIF_SA to be simultaneously acquired, providing an opportunity to verify how each affects lumbar vertebral perfusion quantification. Nevertheless, selection of an axial slice has two drawbacks. First, only one vertebra can be viewed in each slice. To pursue a high temporal resolution of 2 s per dynamic phase, we acquired only two axial slices. When additional lumbar vertebrae are examined, lower temporal resolution and a longer acquisition time will be encountered when using axial slices. Sagittal and coronal slices allow the capture of multiple vertebrae in a single slice; however, they cannot provide the AIF_A and AIF_SA simultaneously. Notably, when scoliosis or malalignment of the lumbar spine is encountered, an attempt to acquire multiple vertebrae via sagittal or coronal slices, respectively, could result in limited success. Second, measures of AIF_SA on axial slices are susceptible to partial volume effects^40^ and in-flow effects^41^. A thin slice can reduce the partial volume effects; nevertheless, a slice thickness of 10 mm provides a greater SNR and a chance to include the entire course of the curving segmental artery in a single axial slice. To eliminate the in-flow pulsation on AIF_A, a pre-saturation band with parallel slices was applied on the upstream aorta. However, this pre-saturation band might also affect the calculation of peak concentration of the AIF, requiring further verification when applied.

Parker's model with a population-based flow-adapted fixed model and a correction AIF method was used as a reference^25,30,42^. The AIFs were selected individually for each participant, then the peak concentration in the blood (*C*_*b*_(t)) of the aorta was adjusted to 6 mmol in the population-based Parker model^25^. Simulation results show that certain perfusion parameters are sensitive to the peak of contrast concentration in the blood. A comparison between three AIFs (AIF_PA, AIF_A, and AIF_SA) in vivo shows that all values for *K*^*trans*^ and *v*_*e*_ are significantly greater using AIF_SA compared to either AIF_PA or AIF_A. This might be attributable to a greater concentration in AIF_PA and AIF_A compared to AIF_SA. Given the fixed blood volume found in the vertebra, an AIF with a greater peak concentration, as seen in the aorta, will generate a lower blood transfer constant due to a greater blood volume in the aorta. This can explain why *K*^*trans*^ and *v*_*e*_ are lower at greater peak concentrations using AIF_A compared to AIF_SA, where peak concentrations are lower. Simulation results further show that *v*_*p*_ increases as delay time increases, but did not vary based on AIF selection. Differences in the results of simulations compared to human studies might be explained by insufficient time delays between the aorta and the segmental artery in the human studies.

This study has several limitations. First, a sample size of eight is small; however, the appropriate number of healthy volunteers should be carefully deliberated, give the risks of allergy, nephrotoxicity, neurotoxicity and potential nephrogenic systemic fibrosis, which must be considered together. We strictly followed the ethical principle to recruit the smallest number of healthy participants (to receive gadolinium-required DCE-MRI) possible while maintaining optimal power for statistical analysis. We similarly chose a minimal sample of participant for a previous study^20^. This limitation was remedied by data augmentation: we chose two vertebrae from each participant, resulting in a pool of 16 vertebrae to examine. Furthermore, simulation results for perfusion parameters related to the peak concentration in the AIF are consistent with those from our human study. Second, only two lumbar vertebrae were scanned per participant, and only axial views were obtained. While axial images have the disadvantage of limited number of vertebrae examined, they outperform sagittal and coronal images in their ability to allow AIF_A and AIF_SA to be acquired simultaneously. Third, because of the thicker slice thickness than the diameter of segmental artery, partial volume has an effect not only on the AIF_SA theoretically but also the resulting pharmacokinetic parameters. We intentionally chose a slice thickness of 10 mm to increase the SNR in the DCE-MRI at a high temporal resolution of 2 s per dynamic phase and to include the entire course of the segmental artery in a single axial slice, knowingly risking partial volume effects. Problems caused by partial volume effects were partially remedied by applying subtracted DCE-MRI, thus providing dynamic images with improved SNRs that allowed easy detection of the segmental arteries. A single pixel centrally located in the artery and clearly demonstrating the first-pass peak within the ROI encompassing the segmental artery was chosen to fit the AIF_SA. By including data only from the baseline, upslope, peak, and 3 to 4 data points following the peak, only the first pass was used to fit AIF_SA. Nevertheless, further studies using various slice thicknesses are suggested to verify the partial volume effects on the measures of peak enhancement in AIF_SA. Fourth, AIF_A might be inherently influenced by the effects of in-flow enhancement on 2D axial images. Our remedy was to apply a pre-saturation band parallel to the slices of the acquired images on the upstream aorta and used the peak of AIF_PA as a correction. Quantitative evaluations of the in-flow enhancement effects on lumbar vertebral perfusion parameters are warranted. Fifth, we did not measure and did not correct for B1 field inhomogeneity. An uncorrected B1 field inhomogeneity is potentially a biasing factor; however, its influence is location specific, and the effects on perfusion parameters in a given location (e.g., the lumbar vertebrae) is time invariant. Therefore, its influence in a comparative study between AIFs, such as this study, is limited. Nevertheless, further studies would verify its effects on the quantification of lumbar perfusion parameters. Sixth, we enrolled only healthy participants and did not include patients with diseases of the lumbar vertebrae. Inclusion of those with disease was beyond the scope of this study, which was performed to test our hypothesis. In another study, we showed a 14% decrease in blood perfusion between two degenerated disks in the lumbar vertebra compared to two normal disks^11^. A study designed to compare the lumbar vertebral perfusion parameters generated using PWM vs. RBM and using AIF_A vs. AIF_SA has been launched in the environment of degenerative lumbar disease. Finally, we did not evaluate the test–retest reliability nor inter-observer agreement. Further study is warranted to verify the robustness of our findings. Accordingly, the results of human experiments presented in this study were related to the selection of AIF solely based on the axial images. We suggest readers to notice the potential limitations of this study and not over-emphasize the absolute values of presented parameters while interpreting our experimental results.

In conclusion, the perfusion parameters *K*^*trans*^ and *v*_*e*_ in AIF_SA are significantly greater than those in AIF_A. Whether it is beneficial to simultaneously acquire both AIFs to quantify vertebral perfusion when using axial slice orientation deserves further verifying studies.

## Data Availability

The datasets generated during and/or analyzed during the current study are available from the corresponding author on reasonable request.

## Abbreviations

AIF: Arterial input function
AIF_A: Patient-specific aortic AIF
AIF_PA: Population-based aortic AIF
AIF_SA: Patient-specific segmental arterial AIF
DCE-MRI: Dynamic contrast-enhanced magnetic resonance imaging
KWT: Kruskal–Wallis test
PWM: Pixelwise method
RBM: ROI-based method
SA: Segmental artery
SNR: Signal-to-noise ratio
WSRT: Wilcoxon signed rank test
